# The Use of Salicylic Acid in Acute Rheumatism

**Published:** 1878-08

**Authors:** S. W. Gillespie

**Affiliations:** Sterling, Ill.


### THE USE OF SALICYLIC ACID IN ACUTE RHEUMATISM.

By S. W. Gillespie, M. D., Sterling, Ill.

There is, perhaps, no disease, the treatment of which occasions the practitioner so much trouble and anxiety as acute rheumatism. The number of remedies which have been brought forward for its relief, and which have enjoyed a short season of popularity and then fallen into disuetude, is almost unlimited. At the present time the profession throughout the world are testing the merits of salicylic acid and its compounds, as remedies in this disease.

It will be conceded by every one that no two persons obtain exactly similar results from the use of the same drug in cases which resemble each other; but the testimony of a large number of persons who have observed the effects of the same remedy in a certain class of diseases, is of service in determining its therapeutic value.

On looking over the literature upon this subject, and comparing the different reports from various physicians and hospitals, both in this country and in Europe, the following conclusions seem justified:

1st. That in acute rheumatism of moderate severity, salicylic acid and its preparations are powerful remedies.

2nd. That they are of less value in the sub-acute and chronic types, and in those in which there is delirium.

3rd. That in moderate cases the pain is relieved, and the temperature lowered in from two to three days after the commencement of the treatment.

4th. That cardiac complications are prevented, as a rule, though they occasionally occur, as do such symptoms as vomiting, deafness and tinnitus.

5th. That to make the cure permanent, the remedy must be continued four or five days after convalescence is established.

As to the explanation of the action of salicylic acid, very little can be said at present. There is no doubt that its action upon the kidneys is very great, and that by its eliminating power in this direction the temperature is reduced, and the removal of the *materies morbi* effected. That the results of the salicylate treatment are much better than those in which the old remedies are used, will be evident by a comparison of the tabulated cases given hereafter (compiled from reports by various authorities) with cases treated by other remedies. The frequency of administration and dose of the drug is varied by different physicians, but the average is 60 centigrams every two hours. The best combination seems to be with sodic bicarbonate, glycerine and water, as the least stomach trouble has followed its use in this way. In a number of the cases reported, hot applications, such as poultices, mustard draughts and hot fomentations were applied to the swollen limbs, and one physician reported several cases in which the remedial effects of the acid seemed to be increased by the addition to the mixture of liq. potassæ accetatis.

In the cases which I have tabulated, the treatment was with salicylic acid or salicylate of soda, the preference being given to the latter, from the fact of its being more pleasant to the patient and of producing the least amount of stomach trouble. To obtain good effects from salicylic acid, the drug must be pure. Several cases are reported in which dangerous symptoms followed its administration ; and, in one instance, death occurred after it had been used a week. These dangerous symptoms are undoubtedly caused by using an article which contains carbolic acid, this acting as a sedative to the heart's action. So far as is known, no unpleasant symptoms have followed the use of a pure acid.

To give some idea of the value which is placed upon salicylic acid and its preparations in the treatment of acute rheumatism, I have condensed a few reports from a number of practitioners and hospitals.

In the Queen's Hospital, at Birmingham, Dr. Sawyer has treated a number of cases of acute rheumatism with salicylic acid. His former plan of treatment was to place the patients between blankets and administer potassic bicarbonate ; all cardiac troubles he treated with blisters and leeches over the heart, and Dover's powder. In the cases he reports, the pain was relieved and the temperature lowered in from two to three days after the patient had been placed on the acid treatment. The tendency to pericardial troubles was lessened by its use.

Dr. Carter, of Bellevue Hospital, N. Y., reports in the *N. Y. Med. Record* for Oct., 1876, five typical cases of acute rheumatism which he treated with marked benefit with the combination of salicylic acid, sodic bicarbonate, glycerine and water. In another case reported in the same article, a child of six years, in whom the joints seemed to be invaded generally, the pulse being 140, temperature in the axilla, 39° C., the patient was put on this mixture with the result of reducing the pulse to 128, and the temperature to 38.5° C., in the first twenty-four hours, besides lessening the pain and swelling in the joints.

Prof. Germain Sée, of the École de Médecine, Paris, in a late clinical lecture, claims that the action of salicylate of soda in febrile articular rheumatism is simply marvelous. He also finds it useful in the sub-acute and chronic forms. The action of the remedy he is not prepared to explain, but thinks it does not act by its anti-pyretic properties, but by some specific action on the morbid agent which produces the affection; for, besides its sedative action, it has a modifying influence upon the morbid tissues. In his cases the swelling, heat and pain were often removed in forty-eight hours. In a few instances the acid seemed to produce delirium, which ceased as soon as the medicine was suspended. In typhoid fever, M. Sée thinks the administration of this preparation is not only useless but dangerous, from its tendency to hasten perforation of the bowels.

Mr. William Corter, of Liverpool, has had one fatal case of acute rheumatism in which salicylic acid was used, and one in which he thinks death would have taken place had not the cold bath been used. In a number of other cases the acid treatment has been very successful in his hands. In those cases where the temperature runs rapidly up to 41.6° C., he thinks the only hope lies in the cold bath.

Dr. S. J. Holmes (*N. Y. Med. Rec.*) says: "After the employment of salicylic acid in a number of cases, I have found it infallible, furnishing early in each case complete immunity from complications, heat, swelling and pain, and in *no* case where the drug was employed early have I met with cardiac troubles. In one case where its employment was delayed, I got endocarditis, but am satisfied had I employed the remedy earlier, the patient would have escaped this complication. I have employed it in cases of varying extent and severity, and as early as the second day have found my patients free from pain and fever, and an abatement of the swelling of the joints on the third or fourth day. By its continued administration for a few days, I have found no propension to immediate return of the disease."

During the year 1877, salicylic acid was tried as a remedy for acute rheumatism in all the hospitals in Chicago, and with varying success. At Cook County Hospital the uncomplicated cases were generally treated with the acid, combined with liq. ammon. accetatis, glycerine and water, with the results I have condensed in the table below. At St. Joseph's hospital, the acid was administered in an uncombined state, and was found to disagree with the patients in two out of four cases. In other cases marked improvement occurred in two days. At Mercy Hospital, owing to the combination of the acid with potassic bicarbonate, potassic iodide and tincture of stramonium, no conclusion can be arrived at as to its remedial effects. In the hospital of the University of the State of Pennsylvania a number of cases have been treated by the remedy with the best of results, the reduction of temperature being very decided in from two to three hours, with an alleviation of the pain and swelling in two or three days.

As before stated, every one who has used salicylic acid has not met with such good results. Several cases are reported in which it has failed to give relief, and in one or two instances alarming symptoms and even death have followed its use.

Dr. Southey (*Brit. Med. Jour.*, May, '77) says, "while it reduces the temperature and lessens the pain in these cases, it does not prevent cardiac complications." And Dr. J. G. Richardson (*Phil. Med. Times*, May, '76) reports four cases in which the use of salicylic acid was followed by adverse symptoms. In one of these the tendency toward alarming prostration was decided. Dr. Watelet (*Bull. de Thérap.*) details two cases of acute rheumatism, in one of which gangrene of the lower extremities, and in both severe cystitis followed the use of salicylic acid.

Dr. C. H. Hall, Macon, Ga., reports (*Phil. Med. and Surg. Jour.*, Jan., '78) one case of death, and two others in which dangerous symptoms occurred after its use. His first case, a colored woman of twenty years, was taken with acute rheumatism in the joints of her right arm, fever moderate. On the disease extending to the left arm and limbs, he prescribed salicylic acid in doses of three decigrams every two hours. Constant amelioration of the pain, fever and swelling occurred, until he pronounced her convalescent on the seventh day. At daylight on the morning of the eighth day he was suddenly called, and found her dead. For half an hour previous to her death, she had complained of great pain in the region of the heart, and difficulty of breathing. His second case was that of a young man, seventeen years of age, whose heart had been previously affected by rheumatism. When the doctor recognized inflammatory rheumatism, he ordered salicylic acid in three decigram doses every two hours. This relieved the fever, but so increased the heart trouble that on the sixth day he substituted alkalies for the acid mixture, which resulted in an immediate relief of the cardiac trouble, and a good recovery from the disease. The third case was of a similar nature. The salicylic acid producing such cardiac pain that it had to be replaced with anodynes and alkalies. The doctor attributes the increased suffering and death of the first patient to the continued use of the acid. From the fact that these cases followed each other closely, and were in all probability treated with a dose of the same specimen of salicylic acid, it is fair to infer that the preparation used was an impure article.

The following table of cases has been compiled from various sources. All the patients were treated with salicylic acid or its preparations. The only fatal cases which I can find recorded have been referred to :

| NAME. | Number of patients. | Average time patient sick in days. | Ave. time pain was relieved in after treat. commenced. Days. | Ave. time taken to reduce temp. Hours. |
|---|---|---|---|---|
| Dr. Sawyer, Birm., Eng. | 3 | 12 | 4 | 6 |
| Dr. Clark, N. Y. | 5 | 7 | 4 | — |
| Dr. Perrin, Mich. | 3 | 12 | 2 | — |
| Leeds Infirmary | 9 | — | 2 | — |
| Prof. Traube | 14 | — | 3 | 48 |
| Dr. Stricker, Berlin | 14 | — | 2 | — |
| Dr. Jacobhouse | 45 | — | 3 | 24 |
| M. Sée, Paris | 52 | 13 | 3 | 48 |
| Cook Co. Hospital | 18 | 22 | 3 | — |
| St. Joseph's Hospital | 4 | — | 2 | — |
| Other cases | 33 | 16 | 3 | 24 |
| Total | 200 | Ave. 13.5 days. | Ave. 2.8 days. | Ave. 30 hours. |

NOTE.—In the blank places the time was not given.

